# Role of Nerve Growth Factor (NGF) and miRNAs in Epithelial Ovarian Cancer

**DOI:** 10.3390/ijms18030507

**Published:** 2017-02-26

**Authors:** Rocío Retamales-Ortega, Lorena Oróstica, Carolina Vera, Paula Cuevas, Andrea Hernández, Iván Hurtado, Margarita Vega, Carmen Romero

**Affiliations:** 1 Laboratory of Endocrinology and Reproductive Biology, Clinical Hospital University of Chile, Santiago 8380456, Chile; rmretama@gmail.com (R.R.-O.); lorenaorostica@gmail.com (L.O.); carolinavera@ug.uchile.cl (C.V.); paucuevasl.2008@gmail.com (P.C.); andrea.gx24@gmail.com (A.H.); ihurtado@hcuch.cl (I.H.); mvega@hcuch.cl (M.V.); 2 Department of Obstetrics and Gynecology, Clinical Hospital, Faculty of Medicine, University of Chile, Santiago 8380456, Chile; 3 Advanced Center for Chronic Diseases (ACCDiS), Santiago 8380456, Chile

**Keywords:** neurotrophins, nerve growth factor (NGF), Tyrosine kinase A receptor (TRKA), epithelial ovarian cancer, microRNAs

## Abstract

Ovarian cancer is the eighth most common cancer in women worldwide, and epithelial ovarian cancer (EOC) represents 90% of cases. Nerve growth factor (NGF) and its high affinity receptor tyrosine kinase A receptor (TRKA) have been associated with the development of several types of cancer, including EOC; both NGF and TRKA levels are elevated in this pathology. EOC presents high angiogenesis and several molecules have been reported to induce this process. NGF increases angiogenesis through its TRKA receptor on endothelial cells, and by indirectly inducing vascular endothelial growth factor expression. Other molecules controlled by NGF include ciclooxigenase-2, disintegrin and metalloproteinase domain-containing protein 17 (ADAM17) and calreticulin (CRT), proteins involved in crucial processes needed for EOC progression. These molecules could be modified through microRNA regulation, which could be regulated by NGF. MicroRNAs are the widest family of non-coding RNAs; they bind to 3′-UTR of mRNAs to inhibit their translation, to deadenilate or to degraded them. In EOC, a deregulation in microRNA expression has been described, including alterations of miR-200 family, cluster-17-92, and miR-23b, among others. Since the NGF-microRNA relationship in pathologies has not been studied, this review proposes that some microRNAs could be associated with NGF/TRKA activation, modifying protein levels needed for EOC progression.

## 1. Introduction

Ovarian cancer is a deadly disease that causes over 140,000 deaths every year [1]. Given the lack of specific symptoms and the poor efficacy of the currently available treatments, the survival rate remains below 50% overall 5-year [2]. Nerve growth factor (NGF) and its high affinity receptor, Tyrosine kinase A receptor (TRKA), are overexpressed in ovarian cancer and they have been associated with increased proliferation, survival and angiogenesis [3].

NGF, through TRKA activation, can alter the expression of several molecules associated with cancer development and progression [3]. There is also evidence to suggest that NGF could control the expression of microRNAs (miRs) [4,5]. miRs are small non-coding RNA molecules that downregulate gene expression by acting on mRNAs [6]. Several miRs have been linked to cancer, either through overexpression or downregulation, altering the levels of oncogenes or tumor suppressor genes [7].

In this review, an overview is given about ovarian cancer and its most common form, epithelial ovarian cancer (EOC). A summary of neurotrophins and miRs roles on EOC is also provided. Finally, a potential link between EOC, NGF and miRs is established.

## 2. Ovarian Cancer

Ovarian cancer remains major health problem worldwide, with over 225,000 new cases and 140,000 deaths reported annually [1]. Symptoms associated with ovarian cancer are often nonspecific [8]; therefore, the majority of patients are diagnosed at advanced stages of the disease [9], which increases treatment cost and diminishes survival rate [10].

It has been suggested that ovarian cancer is linked to an increased number of menstrual cycles [11]; early menarche, null parity, late menopause and not taking oral contraceptives are among its risk factors [12]. Other risk factors include smoking [13], obesity [14] and estrogen-based hormone replacement therapy after menopause [15].

A dualistic model categorizes ovarian tumors into two groups: type I and type II [16]. Type I tumors are part of a morphological continuum of tumor progression starting with benign tumors that develop into borderline tumors and finally into invasive tumors. These invasive tumors lose their differentiation along with their progression. Type II tumors, on the other hand, are aggressive high-grade, poorly differentiated carcinomas that are usually diagnosed at an advanced stage and have poor prognosis [16]. Clinically, ovarian cancer is classified according to the International Federation of Gynecology and Obstetrics (FIGO) criteria [17]. According to these guidelines, stage I consists of a tumor that is limited to the ovaries, while stage II tumors have expanded to the peritoneal space. In stage III, the tumor has spread through one or both ovaries, accompanied by primary peritoneal cancer and metastasis to the retroperitoneal lymph nodes. Lastly, stage IV consists of distant metastasis to extra-abdominal organs [17].

For ovarian cancer, the 5-year survival rate is low [2]. Because of the lack of efficient early diagnosis tools, ovarian cancer is mostly diagnosed at advanced stages, as has already been stated. This is unfortunate, given that early detection is the most effective means of reducing ovarian cancer mortality [18]. For example, currently 25% of ovarian cancer cases are detected at stage I or II [9]; however, if 75% of ovarian cancer cases were to be detected at an early stage of the disease, the number of deaths would be reduced by 50% [19]. At present, only 15% of patients are diagnosed when the disease is confined to the ovary at the time of diagnosis [9]; in most cases, at the time of diagnosis the disease has already spread through the ovary and into the peritoneal space. Under these conditions ovarian cancer responds poorly to therapy and the 5-year survival rate drops to between 17% and 30% [20]. However, if diagnosed at an early stage, when the disease is restricted to the ovary, about 90% of patients survive 5 years after treatment [21]. The late diagnosis that characterizes ovarian cancer is in part due to the lack of specific symptoms, which include abdominal pain, fatigue, indigestion, constipation, back pain, menstrual irregularities and changes in appetite [22].

The main treatment for ovarian cancer is surgery followed by chemotherapy [23]. The surgery consists of cytoreduction in order to remove the tumor, and then chemotherapy is applied to eliminate the remaining tumor mass [24]. First line chemotherapy involves the use of platinum-based therapy, including cisplatin and carboplatin, with an adjuvant therapy like paclitaxel [25]. Around 70% of patients respond to this combination, making ovarian cancer a highly chemotherapy-sensible cancer [26]. However, approximately 60% of patients develop recurrence, and in these patients the returning tumor is resistant to the first line treatment [27]. Second- and third-line treatments usually do not have a high response rate, decreasing the 5-year survival rate to 27% [28].

Because the late diagnosis and poor response to treatment, ovarian cancer ranks among the leading causes of cancer death, being the eighth most common cause of mortality in women due to cancer worldwide [29]. Even though extensive research has been done in order to better diagnose and treatment for this disease, ovarian carcinoma pathogenesis is not yet completely understood.

## 3. Epithelial Ovarian Cancer

Ovarian cancer consists of a malignant tumor that forms in the ovary. The ovary has three main cell types that can develop into a different type of tumor: germ cells can grow into germ cell tumors [30], stromal cells give rise to stromal sex cord tumors [31] and the origin of EOC is from the epithelial cells of the ovary [32]. Most malignant tumors are of epithelial origin, comprising around 90% of all ovarian cancers [33].

About 10% to 20% of EOC cases are of hereditary origin [34], and mutations of the breast cancer gene 1 (BRCA1) or the breast cancer gene 2 (BRCA2) are the most significant genetic mutations associated with an increase in overall ovarian cancer risk [35]. EOC is more common in older, postmenauposal women; therefore, this tumor is usually found in inactive ovaries that are no longer undergoing reproductive cycles [36].

While it was originally thought that EOC originated from the epithelial cells that can be found on the outer layer of tissue surrounding the inactive ovary or on the surface of ovarian cysts [32], there are presently several theories relating to the cell origin of EOC. Some investigators propose that EOC could develop from cysts located in the secondary Müllerian system [37]. According to this theory, the tumor would grow from these cysts and thus appear to have an ovarian origin. Also, recent research suggests that EOC arises at the fallopian epithelium and it spreads to the ovary from there [38].

In EOC, just like in any cancer, several signaling pathways are altered, resulting in uncontrolled growth, apoptosis avoidance and the acquisition of invasion capability, among other carcinogenic features [39]. One of the most studied altered mechanisms in EOC is angiogenesis, a necessary process given the large size that characterizes ovarian tumors [40]. Vascular endothelial growth factor (VEGF) is an angiogenic factor and potent mitogen for the vascular endothelium [41] and one of the most important factors in ovarian angiogenesis, both in normal [42] and in pathological tissues [43]. Bevacizumab, a monoclonal antibody targeting VEGF, has shown moderate success in clinical trials against ovarian cancer [44,45] and it has been approved in Europe against advanced or recurrent in this disease in combination with other chemotherapy medicines [46].

In addition to the importance of angiogenesis in tumor processes, cancer cells are characterized by a lack of cell growth control [39]. This occurs in part through signaling generated from a variety of growth factor receptors, including the ones belonging to the tyrosine kinase receptor family [47]. Neurotrophins can interact with these receptors, inducing pro carcinogenic responses [48].

## 4. Neurotrophins and Their Role in Ovarian Cancer

Neurotrophins are a family of small polypeptides growth factors that consists of five members: NGF, brain-derived neurotrophic factor (BDNF), neurotrophin 3 (NT-3), neurotrophin 4/5 (NT-4/5) and neurotrophin 6 (NT-6) [49]. Neurotrophins act by interacting with two different types of receptors: p75 and the TRK members of the tyrosine kinase receptor superfamily [50]. The p75 receptor is able to bind all neurotrophins with low affinity [51], while different TRK receptors bind to a specific neurotrophin with high affinity: NGF binds to TRKA; BDNF and NT4/5 bind to TRKB and NT-3 binds to TRKC, initiating an intracellular signaling response [52].

NGF was the first neurotrophin to be described [53]. It was discovered in the nervous system; however, it has been found to have functions in several other systems, including the cardiovascular [54], endocrine [55], immune [55] and reproductive systems [56]. Neurotrophins and their receptors are involved in the development and normal functioning of the ovary: NGF plays a role in early follicular development by allowing differentiation of primordial follicles into follicles [57]. This function is apparently due to NGF’s ability to act in granulosa and theca cells and to induce their proliferation [58,59]. NGF also participates in ovulation by inducing the release of prostaglandins, a necessary step in the ovulatory molecular cascade [60]. Besides, NGF is able to induce the expression of Follicle Stimulating Hormone Receptor (FSH-R) and estradiol in human granulosa cells [61].

Angiogenesis is a key process during the normal ovarian cycle, due to the need of new blood vessel formation in each ovulatory cycle [62]. In fact, the ovary is one of the few organs in which angiogenesis occurs in a cyclically controlled manner. During reproductive life, VEGF is one of the most important proangiogenic factors involved in cyclic angiogenesis, by being participant in the periodic growth of follicles and in the development [63] and maintenance of the corpeus luteus [64,65]. This growth factor expression is controlled by several factors, including FSH and Luteinizing Hormone (LH), through their receptors FSHR and LHR, respectively [66]. Both NGF and TRKA, its high affinity receptor, are expressed in the ovary [3]. Equal to VEGF, TRKA and NGF are expressed before ovulation, suggesting a role of these proteins in ovarian function and angiogenesis [3]. Interestingly, NGF can induce angiogenesis in endothelial cells [67]. Besides, NGF is able to induce a neovascularization process in the superior ganglion of newborn rats, which is accompanied by an increase of VEGF production [68]. In granulosa cell lines, NGF is able to induce VEGF expression through TRKA activation [69]. These findings suggest that NGF and TRKA are important in the regulation of the normal ovarian function.

**Figure 1:**
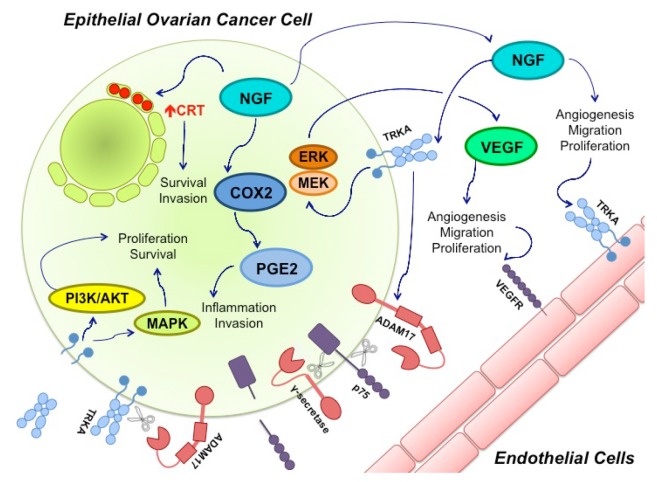
Nerve growth factor (NGF) and its Tyrosine Kinase A receptor (TRKA) are involved in several signaling pathways in epithelial ovarian cancer (EOC). Ovarian cancer cells express and secrete NGF. Through TRKA activation, NGF induces angiogenesis by directly stimulating endothelial cell proliferation and migration. NGF also regulates angiogenesis in an indirect manner through vascular endothelial growth factor (VEGF) production by epithelial cancer cells. This mechanism involves TRKA activation and the Mitogen-activated protein kinase/Extracellular signal-regulated kinase (MEK/ERK) signaling pathway. Besides, NGF increases ciclooxigenase-2 (COX-2) levels, a proinflamatory molecule that induces protaglandine-2 (PGE-2) production; COX-2 and PGE-2 have been associated with invasion in cancer cells. NGF can also increase calreticulin (CRT) levels (Red Arrow), an endoplasmic reticulum resident whose levels have been found to be elevated in several cancers, where it participates in cell survival and invasion. Disintegrin and metalloproteinase domain-containing protein 17 (ADAM17), a proteinase, also seems to be regulated by NGF-TRKA activation. ADAM17 cleaves the p75 receptor, which is then cleaved a second time by γ-secretase. The biological effects of these cuts remain unknown. ADAM 17 can also cleave TRKA, leaving its intracellular domain active, which contributes to cancer progression through the MAPKs and PI3K/Akt pathways.

In EOC, angiogenesis, which is tightly controlled during the regular cycle, becomes unregulated [70]. Because of the normal angiogenesis that occurs on the cyclic ovary during reproductive life, it is possible to suggest that ovarian cells keep their capability for angiogenesis. After malignant transformation, cancer cells could use their advantage of inducing angiogenesis as a mean to acquire nutrients and oxygen. In line with this, both NGF and TRKA have been involved in the angiogenesis of EOC through VEGF induction [71], whose production is the main component of angiogenesis in ovarian cancer [72] (Figure 1). The VEGF gene encodes five different protein isoforms (VEGF121, VEGF145, VEGF165, VEGF189 and VEGF206) that are generated by alternative splicing from a single gene [73]. While VEGF121 is efficiently secreted from the cell, VEGF165 is partially retained on the cell surface and the other VEGF isoforms are primarily retained on the surface [73]. In EOC explants, NGF induces an increase of VEGF121, VEGF165 and VEGF189 mRNA levels, as well the amount of VEGF protein secreted from the explants; these actions are mediated by TRKA [71].

Neurotrophins have also been involved in other tumorigenic processes besides angiogenesis, including growth deregulation that normally occurs in cancer. Tyrosine kinase receptors are overexpressed in cancer, or have their activity altered [74,75]. As a result, there is an alteration of their intracellular signaling [76]. NGF and TRKA overexpression has been found in several cancer types, including thyroid [77], lung [78], esophagus [79], prostate [75], breast [74] and ovarian cancer [3]. In ovarian cancer, TRKA and its active form, p-TRKA, are overexpressed [80]. Interestingly, studies either have not found p-TRKA in normal tissues or it has only been found in a small number of samples [80]. Besides, NGF induces an increase of proliferation molecules and a decrease of apoptosis markers after NGF stimulation in EOC explants [81] (Figure 1).

NGF appears to be controlling other molecules involved in EOC tumorigenesis (Figure 1), such as ciclooxigenase-2 [82] a molecule that participates in inflammation through prostaglandine E2 production [83]. Disintegrin and metalloproteinase domain-containing protein 17 (ADAM17), a proteinase involved in metastasis and migration [84], also seems to be controlled by NGF [85]. ADAM17 is able to cause TRKA cleavage, facilitating cell survival and proliferation. Besides, upon TRKA activation through NGF binding, ADAM 17 cleaves p75 on its extracellular domain, and afterwards γ-secretase cleaves it on the intracellular domain [86]. The biological consequences of these processes are not completely understood. NGF also induces an increase of calreticulin levels [87], a chaperone that has been associated with survival and migration of cancer cells [88,89]. We are currently doing research to further elucidate these and other pathways in which NGF is involved with EOC carcinogenesis.

The evidence presented above shows that NGF alters the expression of several molecules that are involved in tumorigenic processes in ovarian cancer; however, this deregulation could be through different mechanisms: NGF could act at an epigenetic level, induce DNA mutations, induce chromosomal alterations, and/or alter post-transcriptional or post-translational regulations. Today, one of the most widely studied mechanisms for protein synthesis control is microRNA-dependent post-transcriptional regulation.

## 5. microRNAs and Cancer

miRs are the widest family of non-coding RNAs, their length is ~22 nucleotides and they regulate post-transcriptional mRNAs. In mammals, there have been ~2000 different miRs described, which are conserved in related species [90,91]. miRs are synthesized by RNApol II and they are cleaved, in the nucleus and cytoplasm, for their maturation. miR regulation is performed by the RNA-silencing inducing complex (RISC), composed by a multiprotein complex and the mature miR, where the mRNA target can be paired with partial or total complementarity with miR, leading to mRNA degradation, deadenilation or inhibition of its translation [92,93].

**Figure 2:**
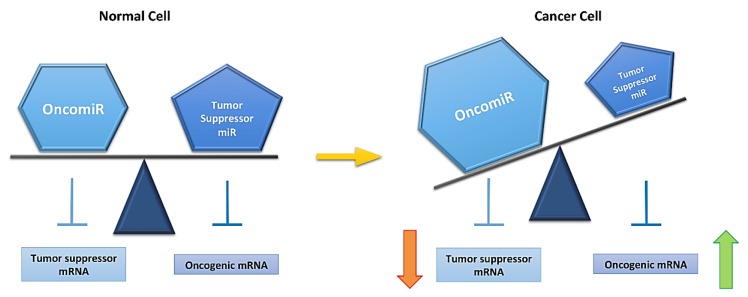
MicroRNA (miR) expression is altered in a cancer cell. A normal cell has equilibrium between oncomiRs (overexpressed miRs in cancer) and tumor suppressor miRs (downregulated miRs in cancer), This equibrium is lost in a cancer cells (yellow arrow). This is reflected on mRNA expression, rising oncogenic and decreasing tumor suppressor mRNA levels, and allowing for cancer development.

miRs have an important role regulating mRNA expression, which reflects on a modification in protein levels. Interestingly, one miR can regulate up to 30 mRNAs and one mRNA can be regulated by several miRs; then, an alteration of miR expression can regulate several processes. A deregulation of miR expression has been found in several pathologies, including glaucoma, neurodegenerative diseases [94], cardiovascular pathologies [95], metabolic diseases [96] and cancer [97], among others. Since deregulation in miR expression is crucial for cancer development [98,99], the roles of miR in this pathology are divided into two groups: oncomiRs and tumor suppressor miRs [100]. OncomiRs regulate mRNA of tumor suppressor genes, while tumor suppressor miRs regulate oncogenic gene mRNA expression. OncomiRs are found to be overexpressed, reducing tumor suppressor gene expression, at the same time that tumor suppressor miRs are found to be downregulated, increasing oncogenic gene expression (Figure 2) [101]. miR expression can be modified in different types of cancer regulating the same mRNAs and producing equal effects in neoplastic transformation. This is a very interesting phenomenon, since different types of cancer could have similar targets that could initiate tumorigenic development [99].

Studies have been done in order to find a miR expression pattern in diverse types of cancer, which is being undertaken both in tissue samples, and in samples that require less invasive methods of extraction, like blood and serum [102]. Even though there is no consensus about miR expression patterns yet [103], some of those that are being studied are breast [104], ovarian [105], esophageal [106], renal [107] and bladder [108] cancers. In ovarian cancer [109], miRs could potentially be used as reliable markers for diagnosis, prognosis [110] and even as therapeutic targets [111].

## 6. Role of miRs in Ovarian Cancer

Several studies have been performed in order to evaluate the role of miRs in ovarian cancer; however, this information has not yet been used with a clinical approach. Some miRs that have been found altered in ovarian cancer include Let-7, miR-200 family, miR-17-92, miR-21, miR-145 and miR-23b [112,113,114]. Most of them have also been studied in other types of cancer, and they can have different roles depending on the cancer type.

In ovarian cancer, Let-7 is a family of tumor suppressor miR, inhibiting a downstream component of the EGFR signaling network (KRAS), regulating cancer-cell proliferation [115], protein that belongs to the non-histone chromosomal high-mobility group (HMGA2) associated with both malignant and benign tumor formation, as well as certain characteristic cancer-promoting mutations [116] and c-Myc [117]; then Let-7 has been identified as a potential maker for early diagnosis [118]. Concerning miR-200 family, it has been reported that miR-200c inhibits protein Zinc finger E-box-binding homeobox 1 (ZEB-1) and miR-200a inhibits ZEB-2 [119], proteins that are important transcription factors that allow epithelial–mesenquimal transition and associated with tumor progression. miR-141 inhibits Keap-1 [120] and miR-200c can regulate B-tubulin III expression [121]. Finally, if miR-200c levels are restored, there is an increase in overall free progression and survival, and it sensitizes cancer cells to cisplatin and paclitaxel therapies [122]. The miR-17-92 cluster, an oncomiR, presents a high frequency of genomic alterations [123] and it induces angiogenesis through inhibition of thrombospondin 1 [124]. miR-21, an oncomir, regulates Phosphatase and tensin homolog (PTEN), this enzyme acts as a tumor suppressor [125], it has an inverse relation with programmed cell death protein 4 (PDCD4) expression [126] and it exerts an inhibitory effect on cancer cell proliferation [127]. This miR has also been associated with recurrence-free survival, which will be lower if miR-21 levels are elevated [128]. miR-145 belongs to the miR143/145 cluster [129]; both miRs have been described as tumor suppressor miRs, and it has been proposed that the downregulation of miR-145 could be used as a predictive value for cancer. miR-145 has been implicated in angiogenesis, decreasing molecules that stimulate this process, like p79S6K1, which regulates hipoxic inducing factor-1a, an important growth factor that stimulates VEGF production, and also regulates directly to VEGF expression [130]. miR-145 also inhibits c-Myc [131], decreasing proliferation and inducing apoptosis. Most studies about miR-145 are focused on miR-145-5p, which is defined as the mature strand, while miR-145-3p is the passenger strand. Interestingly, it has recently been reported that both of these miRs could have biological activity, and it is very rare to find duplex miRs where both strands have biological functions [132]. miR-23b belongs to the miR-23b/27b/24 cluster, it has been described as a tumor suppressor, and some miR-23b targets include cyclin G1 [133] and the transcription factor RUNX2, involved in cellular survival, migration and invasion [134]. In ovarian cancer patients, miR-23b has been found to be downregulated and it has been associated with advanced tumor progression and poor prognosis [133].

## 7. NGF and miRs in Ovarian Cancer

NGF and miR roles in ovarian cancer are well described above, but NGF’s role in miR expression in ovarian cancer is far from well understood. There are some studies regarding miR alteration due to neutrophin action. For example, it was reported that miR-204 upregulation downregulates BDNF in order to enhance anoikis in EOC [135]. With respect to the action of NGF, most of these studies have not been done in EOC or even in cancer; but on neuronal models. What has been reported? Firstly, NGF signaling through ERK protein increases miR-222 and miR-221 levels [4]; secondly, NGF overexpresses miR-221 levels, with consequences in neuronal differentiation [5]; thirdly, NGF raises miR-21 levels, supporting its signaling and protecting cells from neuronal degeneration [136]. While all of these studies have been done on a PC12 cell line, there are also a couple of reports done in bladder [137] and bronchial epithelium [138].

In relation to the signaling pathways that could be involved in NGF-dependent miR expression levels, it has been reported that NGF can stimulate AKT and MAPK phosphorylation in an miR-21-dependent manner, increasing VEGF levels (which are raised with NGF stimulation in EOC). Therefore, miR-21 can be considered as a positive regulator of this neurothrophin signaling [136]. On the other hand, NGF can function as a positive regulator by increasing miR-221/222 through the activation of the ERK1/2 signaling pathway [4]. Another important protein for EOC metastasis is ADAM17, which is regulated by a metalloproteinase inhibitor (TIMP3). miR-222 targets TIMP3, decreasing its levels and allowing ADAM17 expression [139]. Ciclooxigenase-2 (COX-2), an important protein up-regulated in EOC, is regulated by miR-143 [140]. It has been published that this miR is downregulated in EOC [141]; besides, it decreases with NGF stimulation in a cellular line derived from a pheochromocytoma of the rat adrenal medulla (PC12) [5]. Therefore, a NGF-dependent decrease of miR-143 could increase COX-2 levels in EOC.

We are currently studying the downregulation of miR-23b in EOC [133]. As mentioned above, this downregulation coincides with the overexpression of NGF. miR-23b has several validated targets, some of which are shown in Table 1. The table focuses on those that are involved in different processes associated with EOC, NGF or both. miR-23b target genes related with NGF and EOC include proto-oncogene tyrosine-protein kinase (SRC) and a protein that is involved in controlling the activation of RAS/MAPK signaling (SOS1) and super oxide dismutase 1 (SOD1). SRC is a protein involved in TRK signal transduction, SOS1 is a guanine nucleotide exchange factor (GEF) that is also related to TRK transduction, and finally SOD1 is an enzyme that is in charge of converting superoxide radical into hydrogen peroxide, which could enhance DNA damage.

**Figure 3:**
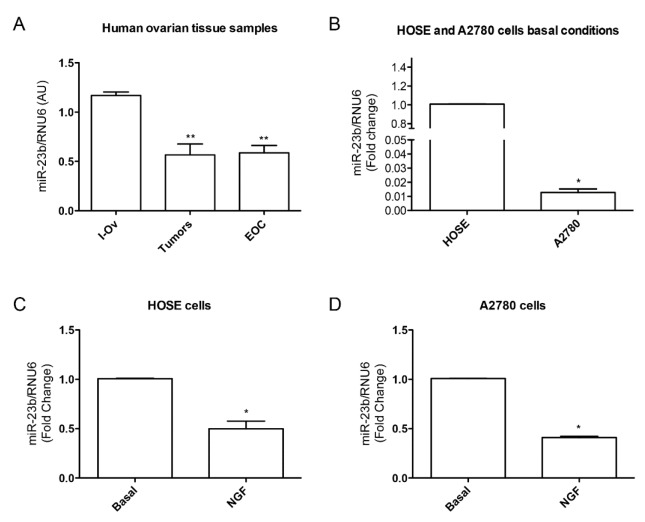
miR-23b levels in epithelial ovarian cancer and its relationship with NGF in cell lines. (**A**) miR-23b levels in tissues from inactive ovary (I-Ov), tumor and EOC samples. miR-23b levels from tumor and EOC samples are significantly lower than I-Ov (** = *p* < 0.01); (**B**) miR-23b basal level comparision between HOSE and A2780 cell lines (* = *p* < 0.05); (**C**) NGF effect on miR-23b levels in HOSE cell line. NGF reduces miR-23b levels respect to basal condition (* = *p* < 0.05); (**D**) NGF effect on miR-23b levels in A2780 cell line. NGF reduces miR-23b levels respect to basal condition (* = *p* < 0.05). Stadistic: Kruskal–Wallis Test: **A**; Mann–Whitney Test: **B**, **C** and **D**.

The evidence presented above leads us to hypothesize that NGF could be regulating miR-23b downregulation, which is an important step for EOC development. To date, as shown in Figure 3, our results have indicated that miR-23b is downregulated during ovarian cancer progression: in tumor and cancer tissue samples miR-23b levels are lower when compared to inactive ovarian tissue samples (Figure 3A, *p* < 0.01). A comparison between basal conditions of A2780 cells (epithelial ovarian cancer cell line) and HOSE cells (epithelial cells from human inactive ovary) show that miR-23b levels are lower in the A2780 cell line (Figure 3B, *p* < 0.05), suggesting that the higher expression of NGF and TRKA in A2780 cell line could affect miR-23b levels. When we evaluated NGF effect on miR-23b expression in both cell lines, we found that stimulation of A2780 with NGF reduces miR-23b levels (Figure 3C, *p* < 0.05), and in HOSE cells a similar effect was found (Figure 3D, *p* < 0.05). Summarizing, these results show that: (a) miR-23b levels are down-regulated in ovarian cancer, as has been reported; and (b) NGF reduces miR-23b levels in HOSE and A2780 cell lines.

The next step is to evaluate which proteins regulated by miR-23b in EOC could be enhancing carcinogenic processes in EOC, and if NGF is regulating expression of these proteins. One example of a miR-23b target and its contribution to EOC development is SRC, an oncogene deregulated in several solid tumors, including ovarian cancer. It is a serine/threonine kinase that activates three signaling pathways: STAT3/MYC, MAPKs and PI3K. While SRC is inactive in normal tissues (when miR-23b presents higher levels), it can be activated and overexpressed in cancer cells, inducing carcinogenic processes such as angiogenesis, proliferation, invasion, motility and chemoresistance. SRC has been found to be elevated in some serous EOC patients, making it a possible therapeutic target [142]. Another example of a mR-23b target is SOS1, which participates in cell migration, a process that requires the participation of proteins from the Rho-GTPase family, including Rac. Rac activity is regulated by Ras through SOS1, and it is responsible for the reorganization of actin cytoskeleton, a necessary process for cell migration. Besides, in ovarian cancer patients, the expression of proteins involved in epithermal growth factor receptor (EGFR) such as a tri-complex SOS1 and Epithermal growth factor receptor Pathway Substrate 8 and an adapter protein ABI-1 (SOS1/EPS8/ABI1) This complex is correlated with advanced clinical stage. In addition, this complex is only present in metastatic ovarian cancer cells, while it is absent in non-metastatic cells [143]. Another molecule involved in EOC development is the transcription factor c-Myc. This factor increases when ovarian cancer cells are stimulated with NGF [81]. Interestingly, it is known that c-Myc decreases miR-23b expression in myeloma [144]. Thus, the negative regulation of miR-23b by c-Myc through NGF could favor EOC progression. The perspectives of these results and analyses are to evaluate the role of NGF in miR-23b regulation in EOC-involved targets.

## 8. Conclusions and Perspectives

**Figure 4:**
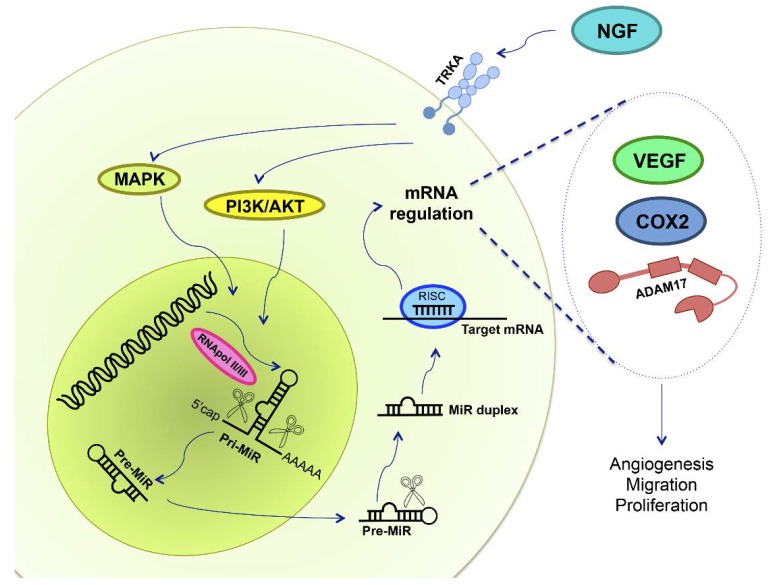
NGF and miRs in epithelial ovarian cancer. NGF binds to its high affinity receptor TRKA, activating MAPK and PI3K/AKT signaling pathways. Deregulation in miR expression has been reported in EOC, and NGF could regulate primary-miR (pri-MiR) synthesis. RNApol II/III synthesizes miRs, and afterwards proteins cleave both pri-miR and precursor miRs (pre-miR), in the nucleus and in the cytoplasm, respectively, leading to miR maturation. mRNA repression is done by RNA-silencing inducing complex (RISC), a multiprotein complex, and by the mature miR, regulating mRNA expression through its degradation, deadenilation or inhibiting its translation. Therefore, we hypothesized that NGF/TRKA modifies miR expression in order to regulate VEGF, COX2 and ADAM17 protein levels. An increase of VEGF, COX2 and ADAM17 are related with changes in angiogenesis, migration and proliferation, processes needed for EOC development.

NGF is a relevant molecule that stimulates epithelial ovarian cancer cell proliferation, migration, invasion and angiogenesis; processes that are crucial for ovarian cancer development. On the other hand, the post-transcriptional regulation of miRs is altered in several pathologies, including EOC, and this deregulation is involved in different characteristics that initiate neoplastic transformation. With respect to how NGF and miR are involved in EOC progression, it is important to evaluate its relationship in this pathology. NGF could modify some miR levels, oncomiRs and tumor suppressor miRs, in order to increase levels of VEGF, COX2, ADAM17, and other proteins (Figure 4). As reported above, it is known that NGF regulates some miR expression in EOC, but it is necessary to further investigate in order to know which miR could be used as an early diagnosis tool and/or for therapy development.

## Figures and Tables

**Table 1:** miR-23b validated targets from miRWalk 2.0.

miR-23b EOC-Related Targets	miR-23b NGF-Related Targets	miR-23b EOC and NGF Related Targets
NOTCH 1	VAV3	SRC
HMGB2	KLF10	SOS1
ZEB1	SOCS6	SOD1
VCAM1	NOTCH1	PTEN
RB1	-	-
CAP1	-	-

## Abbreviations

EOC	Epithelial ovarian cancer
NGF	Nerve growth factor
VEGF	Vascular endothelial growth factor
miR	MicroRNA
TRKA	Tyrosine kinase A receptor
ADAM17	Disintegrin and metalloproteinase domain-containing protein 17
